# Synthesis and Performance Evaluation of Anti-Washout Admixtures for Underwater Non-Dispersive Concrete Based on Nanosilica

**DOI:** 10.3390/ma18112541

**Published:** 2025-05-28

**Authors:** Jian Wang, Kaijian Huang, Hongyan Chu, Jianhui Li

**Affiliations:** College of Civil Engineering, Nanjing Forestry University, Nanjing 210037, China; 17315532694@163.com (J.W.); chuhongyan@njfu.edu.cn (H.C.); subway96@126.com (J.L.)

**Keywords:** underwater non-dispersive concrete, nanosilica, inorganic–organic hybrid polymer, working performance

## Abstract

Anti-washout admixtures (AWAs) are a unique component of underwater non-dispersive concrete (UNDC), which gives the concrete the ability to remain undispersed in water. On some special occasions, freshly mixed underwater non-dispersive concrete is exposed to the erosion of moving water, and conventional acrylamide-based AWAs are only suitable for static water or the water flow rate is small. In this study, the inorganic component nanosilica (NS) is modified, treated, and copolymerized with the organic components acrylamide (AM) and acrylic acid (AA) to form an inorganic–organic hybrid polymer with a hyperbranched structure, which changes the linear structure of the original polyacrylamide molecule, and we optimize the synthesis process. The polymers are characterized at the microscopic level and their compatibility with polycarboxylic acid water-reducing agents (SP) is investigated. In addition, the polymers are compared and evaluated with commonly used PAM in terms of their working performance. The experimental results indicated that under specific process conditions, polymers endow cement mortar with good resistance to water erosion. At the same time, the polymers’ three-dimensional network structure is prominent, with good compatibility with SP and better anti-dispersity. The microstructure of the cement paste with added polymers is dense and flat, but its flowability and setting time are slightly worse. This study provides a new development direction for the development of AWAs under a dynamic water environment, which has specific engineering significance.

## 1. Introduction

Conventional concrete, due to its insufficient viscosity, is easily dispersed by water in underwater environments, which leads to loss of paste, loss of strength, and pollution of the environment. In this context, scholars have developed a kind of underwater non-dispersive concrete, through the addition of anti-washout admixtures (AWAs) [1,2] in concrete, adsorption, and bonding of various components of concrete, to achieve direct pouring in the water, and it is not easy to disperse and segregate, known as “a new generation of underwater engineering materials” [3]. In some special construction areas, such as tail water impact areas of hydroelectric power plants and river channels with certain flow rates, newly poured underwater non-dispersive concrete needs to withstand a certain speed of water flow before hardening. When the water flow speed exceeds the tolerance range of underwater non-dispersive concrete, the surface of the concrete will be eroded by water, which will affect the quality of the construction. Currently used AWAs are only suitable for static water or relatively small water flow environments. Although it is possible to increase the amount of AWAs to improve resistance to scouring, it will cause the concrete to be too viscous, difficult to construct, and have a setting time that is too long, along with other problems [4].

To improve the scour resistance of underwater non-dispersed concrete in water, scholars have made different attempts. Sun et al. [5] gelatinized PAM and explored the flocculation mechanism of gelatinous PAM by the light scattering method. The results showed that the gelation of PAM was conducive to the effective unfolding of the three-dimensional network, which increased the contact area with the cement material and thus effectively enhanced the flocculation ability. In addition, crushing regeneration experiments show that thanks to the special properties of the gel, the gelation of PAM also has a good shear recovery ability. Grzeszczyk et al. [6] added nano-silica and cellulose-based AWAs into underwater non-dispersed concrete and investigated the effect of synergistic effects on the scouring resistance of concrete. It was found that nanosilica further improved the scour resistance of concrete, which was hypothesized to be possible because nanosilica adhered to the three-dimensional network formed by the AWAs, which increased the resistance to migration of cement particles. Some studies [3,7,8,9,10,11] have been conducted to improve the anti-dispersity and mechanical properties of concrete through single or compound mixing of high-fineness materials such as fly ash, silica fume, granulated blast furnace slag, etc., which plays a particular utility. Plant fibers and their products have been shown to improve the compactness of cementitious materials and to increase the strength of the interfacial transition zone (ITZ), presumably contributing to improved scour resistance [12,13]. In addition to changing the concrete material point of view, environmentally friendly self-protective underwater concrete (SPUC) technology provides a new perspective [14,15]. SPUC technology refers to the construction of a water environment through the addition of a kind of underwater protective agent (UPA) to change the characteristics of the water so that the water will not be washed out of the concrete surface of fine particles. Experiments proved that good compressive strength can be obtained in water with a current speed of 0.2–0.5 m/s. The above studies are from the physical level, by changing the state of AWAs, mixing high fineness materials, changing the water medium, and other ways to improve the scour resistance of concrete, and they achieved some results, while the development of specific AWAs for the dynamic water environment of the relevant reports are fewer.

Currently, two types of AWAs commonly used for underwater non-dispersible concrete are the cellulose series and the acrylamide series. The cellulose series [16,17,18,19] will introduce air bubbles to affect the strength in actual use, and the setting time is longer, and the price is relatively expensive. Meanwhile, the acrylamide series [20] has fewer problems and is inexpensive, so it is used more frequently. But in the dynamic water environment, polyacrylamide molecules’ relative linear structure makes their molecular bridge is easy to be destroyed by the water flow, thus causing the concrete surface to be washed away. Additionally, the acrylamide series also has easy agglomeration and other characteristics, affecting the use of efficiency [5]. In the field of oil extraction and wastewater treatment, scholars copolymerize organic components with nanomaterials to obtain polymers with a higher number of molecular branches. Such polymers have better flocculation properties [21,22,23]. In order to improve the usefulness of AWAs in moving water, in this paper, with reference to the above study, an inorganic component, modified nano-silica, was added to the polymerization process of polyacrylamide, to obtain a hyperbranched polymer with nano-silica (NS) as the nucleus and the molecular chain in a stellar structure. It changed the linear structure of the original polyacrylamide molecule. Among the organic monomers, acrylamide and acrylic acid were chosen in the hope of improving the hydrophilicity of the polymer through the water-reducing group (-COOH) in acrylic acid [24]. The focus of this paper will be to investigate whether synthesized hyperbranched polymers can be used as AWAs for cementitious materials and whether the synthesized hyperbranched polymers can impart better scour resistance to cementitious materials as compared to conventional PAMs with linear molecular structure.

## 2. Materials and Methods

### 2.1. Materials

Vinyltriethoxysilane (VTES, AR), acrylamide (AM, AR), acrylic acid (AA, AR), silica (NS, AR), ammonium persulfate ((NH_4_)_2_S_2_O_8_, AR), sodium bisulfite (NaHSO_3_, AR), azobisisobutanimidamide (V-50, 97% purity), EDTA-2Na (AR), sodium formate (HCOONa, 99.5% purity), urea (CO(NH_2_)_2_, AR), 0.1 mol/L NaOH solution, and anhydrous ethanol (C_2_H_5_OH, AR) were purchased from McLean Biochemical Technology Co., Ltd., Shanghai, China. Commercially available anionic polyacrylamide PAM, with a molecular weight of 12 million, were purchased from Shuangjie Chemical Co., Ltd., Henan, China. P.O42.5 cement were purchased from Conch Co., Ltd., Anhui, China, and the composition and content are shown in Table 1. A polycarboxylic acid water-reducing agent (SP) was purchased from Stable Trading Co., Ltd., Nanjing, China, and ISO-standard sand was purchased from Aisio Standard Sand Co., Ltd., Xiamen, China.

### 2.2. Polymer Synthesis

The polymer was synthesized in an aqueous solution using NS as the inorganic monomer and AM/AA as the organic monomer, using a redox–azide composite initiation system, with the following brief steps: The silane coupling agent VTES was weighed and dissolved in an aqueous ethanol solution, and it was stirred with a magnetic stirrer at 880 r/min for 20–30 min to obtain a VTES hydrolysis solution. NS dissolved in ethanol aqueous solution was weighed, ultrasonic treatment for 30 min was applied, and the VTES hydrolyzed solution was slowly added in a dropwise manner. A condensation reflux device was installed, and the reaction was carried out at 70 °C for 6 h. The product was vacuum-extracted, filtered, washed with ethanol, dried, and ground to obtain modified NS (VNS). VNS, AM, and AA were dissolved in deionized water, stirred with a magnetic stirrer at 1100 r/min for 30 min, and ultrasonicated for 30 min. The pH of the aqueous solution was adjusted with NaOH solution, and a small amount of deionized water was added to adjust the total mass concentration of the monomer. Complexing agent EDTA-2Na, chain transfer agent HCOONa, and CO(NH_2_)_2_ were added and stirred with a magnetic stirrer at 1100 r/min for 30 min, and the temperature increased to 35 °C. Nitrogen deoxygenation was continued for 30 min. A dropping funnel was used to slowly add measured redox initiator (NH_4_)_2_S_2_O_8_, NaHSO_3_, and azodyne initiator V-50, stirring evenly, and nitrogen protection was continued. The system was homogenized and continuously protected by nitrogen gas until the system was obviously viscous. The product was purified with anhydrous ethanol and washed repeatedly until whitening and hardness increased, and then it was granulated, dried, and crushed to synthesize the polymer called VNS-AM-AA. The magnetic stirrer (DF-101S) was purchased from Wozhong Instrument Equipment Co., Ltd., Nanjing, China.

**Figure 1:**
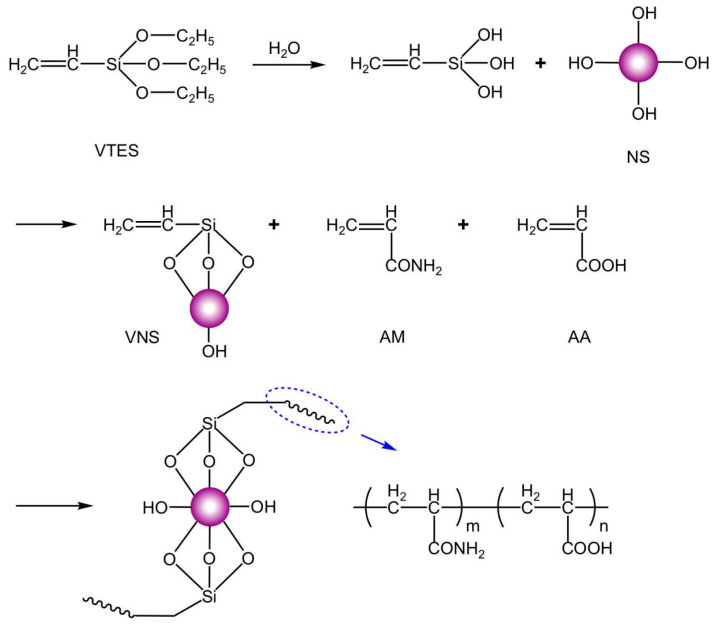
The synthesis principle of VNS-AM-AA.

**Figure 2:**
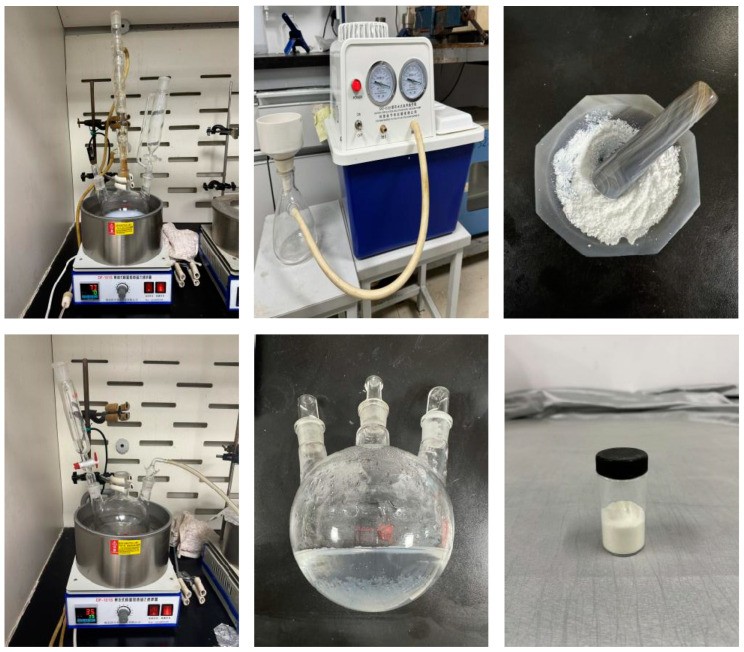
Partial preparation process diagram of VNS-AM-AA.

Based on previous experiments, the optimal synthesis conditions were determined as follows: pH, 7.5; monomer mass fraction of the total reaction system, 26%; initiator mass fraction of the total monomer, 0.2%; molar ratio of (NH_4_)_2_S_2_O_8_ to NaHSO_3_, 1:1; mass ratio of redox initiator to azo initiator, 1:3; 100 mL of water, 6 mg of HCOONa, and 400 mg of CO(NH_2_)_2_; EDTA-2Na, 0.02% of the monomer mass fraction. The chemistry of the synthesized polymers is shown in Figure 1, and part of the preparation flowchart is shown in Figure 2.

The number of OH groups of NS is large, and the number of branched chains grafted on the surface of NS after the reaction is also large, so only a smaller number is shown in Figure 1. VTES is utilized to react with a large number of OH groups on the surface of NS to graft a certain number of double bonds on the surface of NS. After the reaction is completed, it is copolymerized with AM and AA, which will polymerize on the NS surface to form branched chains, and the number of branched chains is determined by the number of double bonds on the NS surface. At the end of the polymerization reaction, a star-shaped molecule with NS as the core and branched chains dispersed around it is formed, i.e., a polymer with a hyperbranched structure.

### 2.3. Polymer Characterization

NS is the core of hyperbranched polymers, and its particle size, degree of modification, and dosage affect the number of branched chains and molecular structure of hyperbranched polymers, thus affecting the flocculation effect. AM contains amide bond, which is the main flocculation group of anti-dispersants, and AA determines the number of anionic charges carried by polymers, so the ratio of AM to AA is very important to study. Therefore, this paper focuses on four synthetic parameters, namely, the particle size of NS, the degree of modification, the amount of addition, and the ratio of organic components, to optimize the flocculation effect of hyperbranched polymers. The particle sizes of NS were selected as 20 nm, 50 nm, and 100 nm, which are commonly used in the market. Modification of NS introduces double bonds and reduces inter-particle agglomeration [25], and the degree of NS modification is controlled by the ratio of VTES to NS.

The apparent viscosity of the cement paste after the addition of synthesized AWA was used as an indicator of the effect of synthesized AWA because of its positive correlation with the resistance to dispersion and yield shear stress, which can reflect the flocculation effect of synthesized AWA to some extent [26]. The apparent viscosity was determined by a rheometer, and the rotor, rotational speed, and test time were kept consistent during the test. The relative molecular mass of the synthesized AWA was calculated from the characteristic viscosity measured by a Wu-type viscometer with reference to the *GB/T 12005.1-1989 Determination for limiting viscosity number of polyacrylamide* [27]. The rheometer (DV3T) was purchased from Brookfield Engineering Laboratories Inc., Middleborough, MA, USA.

FTIR test of the product: The product was dried in a vacuum dryer at 70 °C for 12 h and then mixed homogeneously with potassium bromide in 1:50 and pressed into tablets, which were scanned by infrared spectroscopy using a infrared spectrometer. During the scanning process, the wave number ranged from 4000 to 500 cm^−1^. The vacuum dryer (DZF-6020) was purchased from Suopu Instrument Co., Ltd., Shanghai, China, and the infrared spectrometer (VERTEX 80V) was purchased from Bruker Corporation, Bremen, Germany.

TEM test of AWAs: The AWAs were dried in a vacuum dryer at 70 °C for 12 h. Next, 0.2 g of AWAs was weighed and dissolved in 20 mL of water and stirred with a magnetic stirrer at 880 r/min for 20 min. After stirring, a portion of the product was sucked up and uniformly applied on a copper mesh, and then the sample was made by vacuum drying at 80 °C for 12 h. The samples were tested with a transmission electron microscope to observe the structure and morphology distribution of the AWAs. The accelerating voltage during the test was 100 kV and the test temperature was 25 °C. The transmission electron microscope (JEM-1400) was purchased from Nippon Electric Corporation, Tokyo, Japan.

SEM test of AWAs: The AWAs were vacuum dried at 70 °C for 12 h before sample preparation. An aqueous solution of AWAs with a concentration of 10 g/L was prepared by mixing the AWAs with water and stirring with a magnetic stirrer at 880 r/min for 20 min. The solution was spread on a silicon wafer by sucking up a portion of the solution and dried under vacuum at 80 °C for 12 h. Then, they were subsequently sprayed with gold to produce the samples. The morphological characteristics were observed with a scanning electron microscope. The accelerating voltage was 10 kV and the testing temperature was 20–25 °C. The scanning electron microscope (Quanta 200) was purchased from Focus Electron Ion Co., Hillsboro, OR, USA.

SEM testing of cement paste: After 7 days of curing, the cement paste blended with the two AWAs, the samples were crushed, the internal fragments were taken, and the hydration was terminated with anhydrous ethanol. The fragments were then dried in an oven at 40 °C for 24 h and adhered to a conductive adhesive. After a gold spraying treatment, the surface morphology was observed with a scanning electron microscope. The accelerating voltage during the test was 3 kV, and the test temperature was 20–25 °C. The PAM was incorporated by 1% and VNS-AM-AA by 0.4% to ensure that the cement paste has both good anti-dispersity and flowability. The scanning electron microscope (Gemini 360) was purchased from Carl Zeiss AG, Oberkochen, Germany.

### 2.4. Preparation of Cement Samples

Before the test, a certain amount of AWAs was slowly added to water and stirred until completely dissolved to make a spare AWA solution. The solid materials of the cement slurry were mixed according to the designed weight: firstly, dry mixing was performed manually for 30 s, and then the AWA solution was added and mixed with a mixer at a slow speed for 2 min, followed by fast mixing for 1 min. Table 2 shows the material proportions used in the cement paste, and Table 3 shows the material proportions used in the cement mortar. The unit of the AWAs and the polycarboxylic acid water-reducing agent (SP) was the percentage of the mass of the cement.

### 2.5. Cement Materials Performance Testing

#### 2.5.1. Basic Properties of Cement Paste

This article evaluates whether hyperbranched polymers can be used as AWAs, mainly by measuring the following properties: compatibility between polymers and water-reducing agents; the anti-dispersity, flowability, and setting time of cement materials after adding polymers. This article also tested the relevant performance of PAM for comparison.

Anti-dispersity test: Anti-dispersity is one of the most important properties of underwater non-dispersible cement materials [1], and the strength of anti-dispersity directly determines the quality of construction, so this study tested the pH and turbidity of water samples to characterize the anti-dispersive effect of PAM and VNS-AM-AA. Put 800 mL of water in a 1000 mL beaker, pour 100 mL of freshly mixed cement paste slowly into the beaker, and let it stand for 3 min. Take the upper layer of the clear liquid and measure its turbidity with a turbidimeter (ZD-10A) purchased from Qiwei Instrument Co., Ltd., Hangzhou, China and its pH value with a pen-type pH meter (PH818) purchased from Wan Chuang Electronics Mfg. Co., Ltd., Dongguan, China in order to measure the anti-dispersity of PAM and VNS-AM-AA. The turbidity value of the water sample of the cement paste without the addition of anti-washout admixtures exceeded the range of the turbidimeter, so it is not presented in the article. 

Flowability test: Flowability affects the working performance of underwater non-dispersible cement materials during actual construction, and poorer fluidity will have the phenomenon of difficult pouring and difficult self-compacting and self-leveling. Referring to the *GB/T 8077—2012 Methods for testing uniformity of concrete admixture* [28], the extensibility was measured to gauge the flowability of the net cement paste after the addition of PAM and VNS-AM-AA.

Compatibility test: The relationship between the dispersibility and fluidity of underwater non-dispersible cement materials is contradictory: an increase in dispersibility will cause a decrease in fluidity performance. In practice, we used the blending of water-reducing agents to balance the two properties, so it is indispensable to study the compatibility of synthetic AWAs and water-reducing agents [16]. It has been found that cellulose-based anti-dispersants will react with naphthalene-based water-reducing agents and melamine-based water-reducing agents in alkaline environments, affecting their respective effectiveness [29]. Whereas polycarboxylic acid water-reducing agents (SP) are more compatible with acrylamide-based AWAs, with the conventional dosage of the water-reducing agent ranging from 0.5% to 1.5% of the cement mass [30]. In this experiment, we chose to explore the compatibility of VNS-AM-AA with SP, and the dosage of VNS-AM-AA was fixed at 0.3%. The compatibility of the AWAs with the water-reducing agents was mainly analyzed through the measurement of the cement material’s anti-dispersibility, fluidity, and the loss of fluidity through the time to judge.

Setting time test: Underwater non-dispersible materials may be eroded by water before they are fully hardened, and this erosion is more intense when the water flow rate is faster, so it is not easy for the setting time to be too long. Referring to *JTG 3420-2020 Testing methods of cement and concrete for highway engineering* [31], the effects of PAM and VNS-AM-AA on the initial and final setting time of cement paste were measured.

#### 2.5.2. Scour Resistance of Cement Mortar

Measuring the pH and turbidity of the upper layer of clear liquid in cement mortar after passing through the water layer can somewhat respond to its dispersion resistance when passing through water, but it cannot respond to the surface of the specimen sheared by variable-speed water flow in actual construction. Most projects use underwater non-dispersive mortar or concrete for operation, and the direct study of the scour resistance of cement paste ignores the aggregate components in the actual project. Cement mortar can be regarded as a special kind of fine aggregate concrete in terms of composition, and its rheological properties can directly reflect the rheological properties of concrete. So, in order to further fit the actual engineering, the choice of formulating cement mortar was taken to further study the scour resistance.

It has been shown that the correlation coefficient between the Bingham model and fresh mortar is a good fit and can reflect the rheological properties of cement mortar [32,33,34]. The action process of moving water on cement mortar can be explained by Bingham’s rheological model [35,36,37]. The frictional resistance between the components of the cement paste and the bonding force combine to determine the yield shear stress that prevents the flow and deformation of the paste when it is washed by the water flow. When the shear force of moving water on the cement mortar is greater than the yield shear stress of the paste itself, the surface of the cement mortar will be peeled off by the water, and scouring will occur. Therefore, the size of yield shear stress is the key to determining the ability of cement mortar to withstand water scouring. When the materials used in the cement mortar body are a certain ratio, the frictional resistance between the components and the bond is relatively fixed, and at this time, the size of the shear stress inside the paste body is mainly related to the AWAs increasing the bond. So, the effect of the AWAs directly affects the scouring performance of the concrete. In order to characterize the water scouring resistance of cement mortar after the addition of PAM or VNS-AM-AA, the yield shear stress of cement mortar was measured by a rheometer which was the same as the one previously mentioned. The yield shear stress of the mortar was too small when no anti-washout admixture was added. A rotor change was required to measure these data, so they are not presented in the article.

## 3. Results and Discussion

### 3.1. Optimization of Process Conditions for VNS-AM-AA

**Figure 3:**
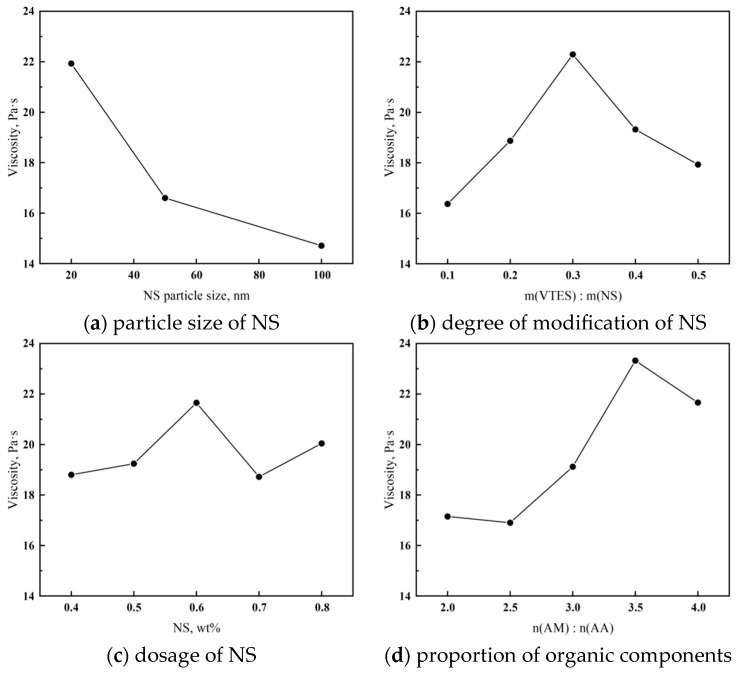
Effects of synthesis conditions on the apparent viscosity of cement paste.

A one-factor method was used to determine the optimal synthesis process by using the apparent viscosity of the cement paste as an index and sequentially investigating the effects of the particle size of NS, the degree of modification of NS, the dosage of NS, and the proportion of organic components on the viscosity-increasing effect of the synthesized polymers. The test results are shown in Figure 3.

Figure 3a shows that the apparent viscosity of the cement is highest at a particle NS size of 20 nm, which indicates the best viscosity enhancement effect of the synthesized polymer. This is because when the particle size of NS is small, the grafted branched chains are more easily unfolded on its surface, which improves the efficiency of the polymerization reaction. So, it results in a higher molecular weight of the synthesized product and a better flocculation effect.

From Figure 3b, it can be seen that the apparent viscosity of the cement paste increases and then decreases with the increase in the degree of modification of NS. And the degree of modification of NS is most suitable for enhancing the viscosity enhancement effect of the polymer when the mass ratio of VTES to NS is 0.3. This is because the higher the degree of modification of NS, the higher the number of branched chains, forming an obvious three-dimensional spatial mesh structure, and the polymer molecules have a large specific surface area. And the contact area with the cement or other components is larger, so the bridging and binding ability is stronger. However, when the modification degree of NS is higher, the number of polymer chains is too high. And the spatial site resistance of the molecules becomes larger, resulting in a shorter length and smaller molecular weight of the synthesized polymer molecules, so the flocculation effect is not good [38]. Moreover, the modification process of NS will lose the hydrogen bond on the surface and increase the hydrophobicity. So, too high a degree of modification will also affect the hydrophilicity of the polymer, resulting in the polymer not being able to dissolve completely, thus affecting the use.

Figure 3c shows that the apparent viscosity of cement paste is the highest when the NS dosage is 0.6% of the total monomer mass. It was analyzed that NS molecules were connected with most of the branched chains, and the number of branched chains increased when its dosage was increased. So, the viscosity-increasing ability was enhanced. NS, on the other hand, leads to partial agglomeration when it is overdoped due to its partial hydrogen bonding, which affects the synthesis efficiency of the polymerization reaction [21].

As can be seen from Figure 3d, the apparent viscosity is maximum when the molar ratio of AM to AA is 3.5:1. The activity of AM is greater than that of AA in the polymerization reaction, so an increase in AM will lead to an increase in the reaction efficiency, which is beneficial to the generation of polymers. But too much AM will lead to an increase in the number of reactive centers, which increases the chance of collision of free radicals and makes the rate of the chain-termination reaction too fast. Therefore, the molecular weight of the polymer product is small, which affects the flocculation effect of the final product [39].

In summary, when 20 nm NS was selected, 30% of its mass of silane coupling agent VTES was put into the modification, 0.6% of the total mass of the monomer was added to join the polymerization reaction, and the molar ratio of the organic component AM to AA was 3.5:1, the synthesized polymer had a good flocculation effect. At this time, the relative molecular weight was about 6 million.

### 3.2. Characterization of VNS-AM-AA

**Figure 4:**
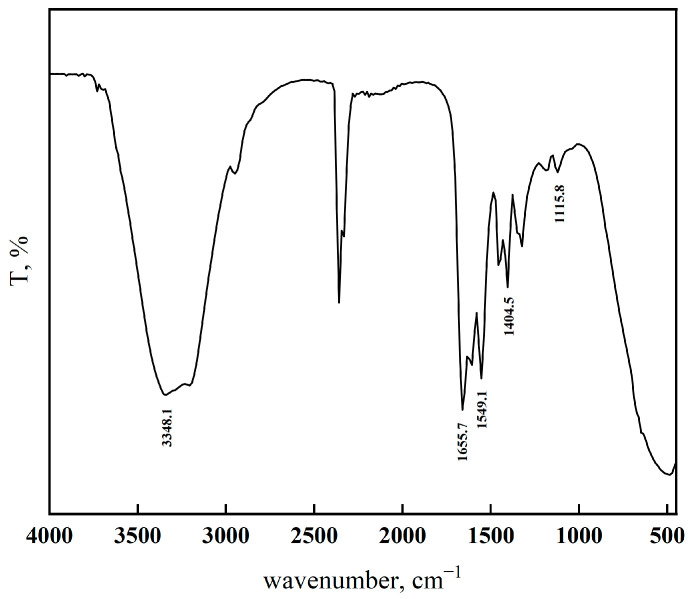
FTIR spectra of VNS-AM-AA.

The synthesized VNS-AM-AA was subjected to FTIR analysis, and the test results are shown in Figure 4. As can be seen in Figure 4, the absorption peak of Si-O-Si bonds is at around 1116 cm^−1^, which represents the successful grafting of NS on the hydrolyzed VTES. The telescopic vibration peak of the primary amide (-NH_2_) is at around 3348 cm^−1^, the absorption peak of the carbonyl group (C=O) occurs at around 1656 cm^−1^, the symmetric telescopic vibration peak of the carboxylic acid group (-COO-) is at 1405 cm^−1^ and around 1549 cm^−1^, and the antisymmetric stretching vibration peak is at around 1549 cm^−1^, which represents the successful copolymerization of AM and AA with modified NS. The IR test results identified the synthesized polymer as the target product.

**Figure 5:**
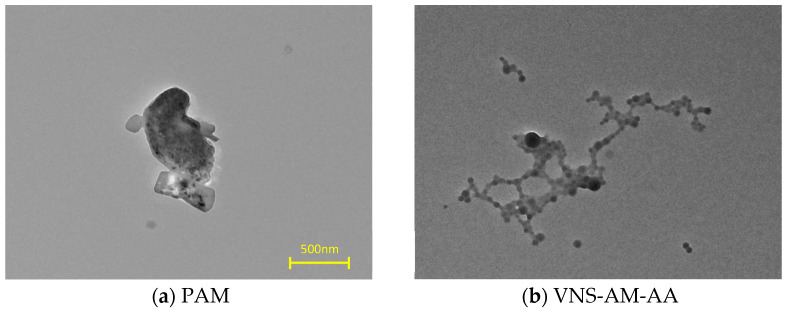
TEM images of PAM and VNS-AM-AA.

TEM images of PAM and VNS-AM-AA are shown in Figure 5. Figure 5a shows the dispersion of PAM in water. It can be found that after the hydrolysis of PAM, the structure is in the form of a thin film, which is difficult to stretch. Therefore, it cannot be well dispersed in the slurry when put into use, and it is often necessary to put in a higher doping amount to achieve the desired performance. In Figure 5b, it can be seen that the polymer as a whole forms NS as the nucleus, connected to the structure of the acrylamide branched chain, and the spatial structure in the water can be effectively expanded. And NS is uniformly dispersed in the matrix with no obvious agglomeration of the phenomenon.

This is due to the modified NS having a certain degree of hydrophobicity. The effect of agglomeration between them is weakened, giving the acrylamide branched chain a certain amount of grafting space. The VNS-AM-AA molecule has a larger number of branched chains, so the molecule has a spatial resistance effect [40]. There is branched-chain grafting in the distribution of uniform modified NS, so that the VNS-AM-AA can be dispersed uniformly in the water and will not produce the effect of agglomeration. The TEM image structure of VNS-AM-AA proves that the degree of modification and dosage parameters of NS are good, and the synthesis conditions of the polymer are feasible.

**Figure 6:**
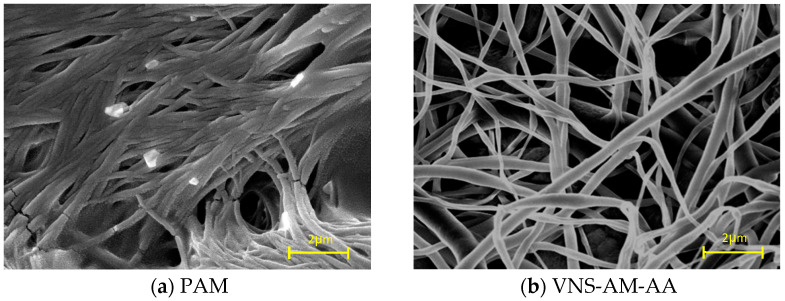
SEM images of PAM and VNS-AM-AA.

The SEM images of PAM and VNS-AM-AA are shown in Figure 6. Figure 6a shows that PAM exhibits a linear shape in water and forms a multilayered near two-dimensional planar laminar structure, whereas Figure 6b shows that VNS-AM-AA forms a three-dimensional mesh structure with a larger specific surface area in water, which has better bridging adsorption properties compared to the laminar structure.

The fact that VNS-AM-AA has a different spatial structure from that of PAM is due to the structure of the combined effect of two factors. On the one hand, the synthesized hyperbranched polymer has a stellar structure with a high number of branched chain bars, which can increase the intermolecular spatial site resistance and prevent the aggregation of macromolecules to form bars along the direction of molecular chains. On the other hand, there are a large number of hydroxyl groups on the molecules of VNS-AM-AA, and these hydroxyl groups will form hydrogen bonds, which will induce the polymer molecules to bridge and ultimately form a mesh-like structure with pores.

### 3.3. Compatibility of Polymers with Water-Reducing Agents

**Figure 7:**
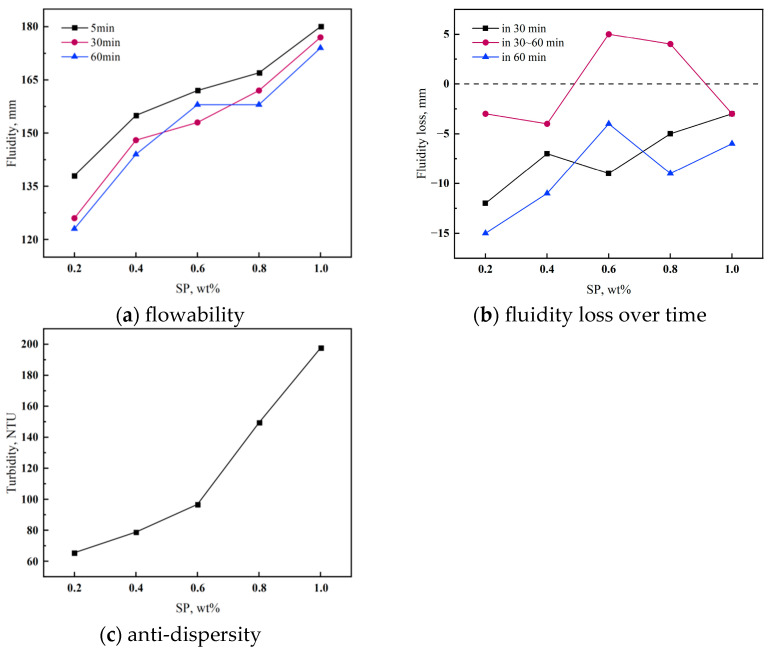
Effects of SP dosage on the performance of cement paste.

The results of the compatibility test of VNS-AM-AA and SP are shown in Figure 7.

From Figure 7a, it can be seen that the initial fluidity of the cement paste increases with the increase in SP, the fluidity is small at 0.2%, the slurry flowability is poor, the change is relatively small when the doping amount is 0.4~0.8%, and it reaches the maximum value of 180 mm at 1%. According to Figure 7c, it can be found that the turbidity value of water samples increases with the increase in SP. The turbidity value is larger at 0.8% to 1%, which indicates that the anti-dispersity is poor at this time. This is due to the fact that the effect of SP in increasing flowability in this interval is greater than the anti-dispersity effect of VNS-AM-AA. As can be seen from Figure 7b, with SP doping at 0.2%, the loss between 30 min and 60 min is relatively large, and at this time VNS-AM-AA plays a major role, while above 0.4%, the fluidity of the degree of loss from 30 min to 60 min is controlled in 10 mm or less. In the 0.4%~0.6% range, the turbidity value is small, and the water samples are relatively clear, which can be seen that the VNS- AM- AA has good compatibility with SP. It can be seen that VNS-AM-AA is more compatible with SP. When the dosage is 0.6% and 0.8%, the fluidity loss from 30 to 60 min is greater than 0, which indicates that SP is still in effect after 30 min. According to the 60 min fluidity loss, the slurry stability was best at 0.6% doping, and 0.6% SP was fixed to be doped in subsequent experiments.

The compatibility of the AWAs and the water-reducing agents depends on whether the two will react. It has been proved that PAM has good compatibility with SP, while the organic component of VNS-AM-AA is similar to PAM, and the inorganic component NS obviously does not react with SP. Therefore, VNS-AM-AA has good compatibility with SP, which is similar to PAM. The good compatibility means VNS-AM-AA’s anti-dispersity and scour resistance can be maintained for a long time, which is favorable for construction.

### 3.4. Anti-Dispersity and Flowability

**Figure 8:**
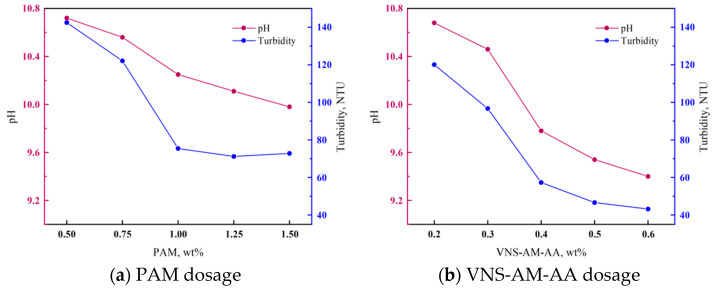
Effects of AWAs type and dosage on anti-dispersity of cement paste.

Figure 8 demonstrates the effect of VNS-AM-AA and PAM on the anti-dispersity of cement paste. As can be seen from Figure 8a, with the PAM dosage from 0.5% to 1.5%, the pH value of the upper layer of the clear liquid continues to decline. After a dosage of 1%, the pH value decreases more slowly, maintained at about 10. Turbidity in dosages of 0.5% to 1% decreased rapidly, reduced by nearly half. In 1% to 1.5%, it stabilized at 70–75 NTU, and at this time, the visual water samples still showed a little turbid phenomenon, but it was not obvious. From Figure 8b, it can be seen that compared with PAM, VNS-AM-AA is more significant in improving the anti-dispersity of cement paste. With its dosage from 0.2% to 0.6%, the pH value of the upper layer of the clear liquid was reduced from 10.7 to 9.4. The turbidity was reduced from 120 to 43 NTU, and it was observed that the water samples were clearer when the dosage was above 0.4%, which indicated that VNS-AM-AA could give the cement paste anti-dispersity at a small dosage. The water samples were still slightly turbid when visualized.

The high efficiency of VNS-AM-AA is because it is easy to spread out in the water and evenly dispersed around the cement paste, while PAM easily forms agglomerates in the water, which makes it difficult to play a full role at higher dosages. A large number of studies have shown that AWAs have the following three main mechanisms [41]: (1) bridging effect: the active functional groups on the AWA molecules will attract and adsorb fine cement particles, and at the same time, the molecules will form bridges by cross-twisting with each other, which connects and wraps the cement particles to form flocs; (2) bridging effect: the substituent groups on the AWA molecules will form a bridge with the cations generated by the hydration of cement, which will react to generate viscous complexes and increase the viscosity of the system; (3) charge neutralization effect: the mobile counter ions in AWA molecules will neutralize the double electric layer on the surface of cement particles, lowering the Z-potential, increasing the attraction potential between cement particles, and making the cement particles easier to agglomerate. The better anti-dispersity property of VNS-AM-AA than that of PAM is due to the fact that the hyperbranched structure contains a higher number of branched chains than that of the linear structure and thus has a stronger binding ability with the cement components. And combined with SEM images, it can be seen that VNS-AM-AA, because of the spatial site resistance effect of the hyperbranched structure, will form a three-dimensional network with a larger specific surface area in the cement paste, which is more favorable for entanglement of cement components than the laminar network of PAM because of the stronger bridge adsorption ability.

**Figure 9:**
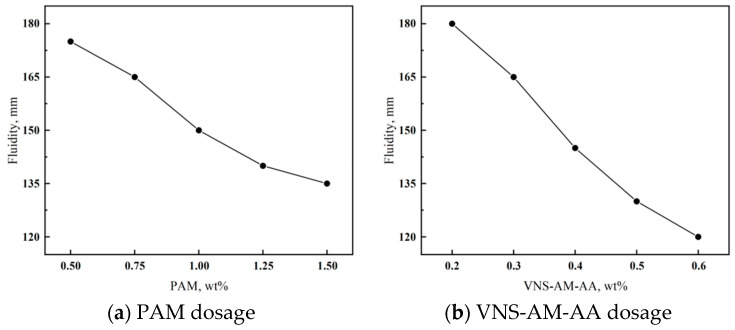
Effects of AWA type and dosage on flowability of cement paste.

Figure 9 shows the difference in the effect of the two AWAs on the flowability of cement paste. With the increase in PAM doping, the fluidity of cement mortar shows a decreasing trend. When the doping amount is 1.25%, the fluidity is 140 mm, and at this time, the cement mortar is more viscous. So, it is not easy for the doping amount of PAM to be more than 1%.

The effect of VNS-AM-AA on flowability generally follows the same trend as that of PAM, but it has a different degree of effect on the flowability of cement paste than that of PAM. On the one hand, the net cement paste achieves similar flowability with less admixture than PAM, and on the other hand, the degree of fluidity decreases by 33% with an increase of only 0.4% in its admixture. However, an increase in the dosage of PAM by 1% resulted in a decrease in fluidity of only 23%. Therefore, compared with PAM, the fluidity of cement mortar is more sensitive to the dosage of VNS-AM-AA. When the dosage of VNS-AM-AA is more than 0.5%, the fluidity of cement mortar is 130 mm and below, and the flowability is poor at this time, which is not enough to satisfy the working performance of actual construction, so it is not easy for the dosage to be more than 0.4%.

### 3.5. Setting Time

**Figure 10:**
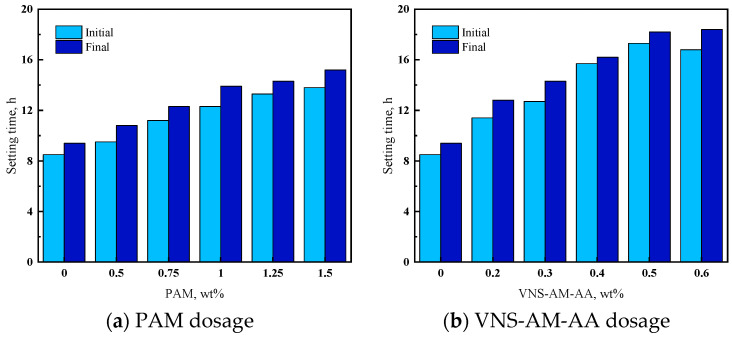
Effects of AWA type and dosage on initial and final setting time of cement paste.

From Figure 10, it can be seen that the setting time shows an overall increasing trend with the increase in AWA dosage, which tends to stabilize after reaching a certain value.

According to Figure 10a, it was found that the initial setting time of PAM was delayed by 1–5 h, and the final setting time was delayed by 1–6 h. Figure 10b found that the initial setting time of VNS-AM-AA was delayed by 3–6 h and the final setting time was delayed by 3–9 h, which were both slightly longer than that of PAM. AWAs would entangle the cement particles and react with the cation to produce a complex, preventing the reaction of cement particles with water [42]. VNS-AM-AA delayed the setting time longer than PAM, which was related to the larger specific surface area of the three-dimensional network formed by VNS-AM-AA and the deeper entanglement of cement particles.

### 3.6. Scour Resistance

**Figure 11:**
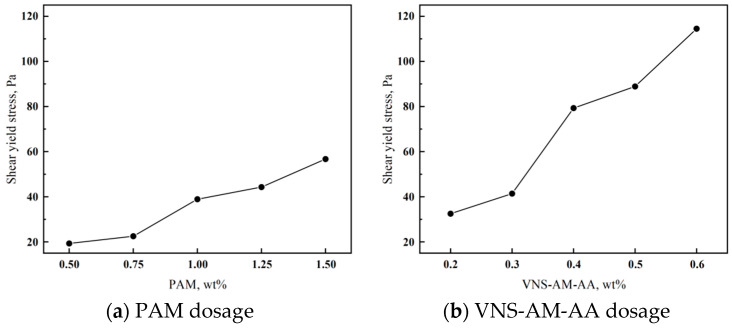
Effects of AWA type and dosage on of cement paste.

As can be seen from Figure 11, the yield shear stress of cement mortar after the incorporation of AWAs increases with the increase in the amount of incorporation. Figure 11a shows that the shear stress is small when PAM is doped from 0.5% to 0.75%, increases relatively rapidly from 1% to 1.5%, and reaches a maximum value at 1.5%. Meanwhile, Figure 11b shows that the shear stress increases rapidly starting from a dosage of 0.4% of VNS-AM-AA, which is greater than that of PAM doped at 1.5%. The shear stress exceeds 100 Pa at 0.6%. It can be seen that VNS-AM-AA can significantly increase the yield shear stress of the cement mortar in comparison with that of PAM, which can improve the performance of the scouring resistance.

The difference in the effect of the two AWAs on the yield shear stress of cement mortar may be due to the difference in their molecular structure. PAM molecules, due to their relatively linear structure, the formation of intermolecular laminar space, and the mortar components connected to the weaker, have a small intermolecular interaction force. In the action of shear, the intermolecular force is easy to be destroyed, and the molecular chain is easy to be fractured from the middle, so even if you add a higher admixture, the yield shear stress is still not high and will lose fluidity. The hyperbranched polymer has more molecular chains, so the molecules are easy to interact with each other, which entangled to form a certain three-dimensional mesh structure. Coupled with a larger number of branched chains, it can produce stronger entangled mortar components. The strength of the 3D mesh structure is higher than that of the laminar structure, so when subjected to shear, the intermolecular forces are stronger and less likely to be destroyed. [22,23]. Due to the unique molecular structure of VNS-AM-AA, the shear force will only act on part of the molecular chain of the polymer, retaining most of the molecular weight of the main molecular chain [21]. So, the yield shear stress is greater than that of mortar mixed with PAM; i.e., the enhancement of the scouring resistance of the cement mortar by VNS-AM-AA is better than that of PAM.

### 3.7. Microscopic Morphology

**Figure 12:**
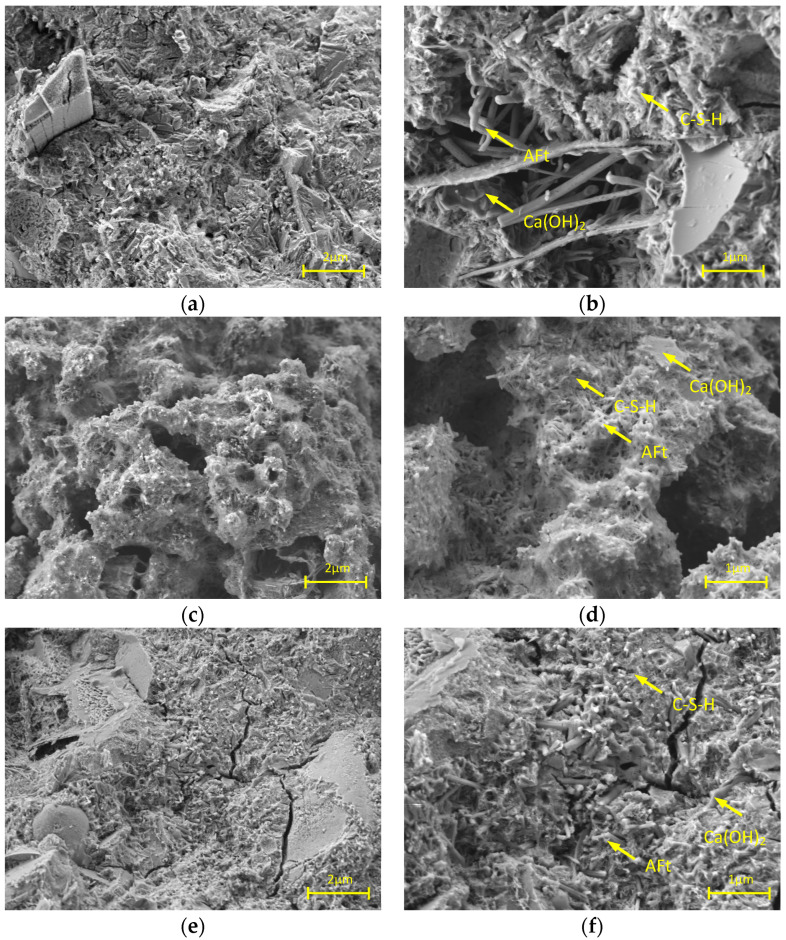
SEM images of cement paste mixed with different AWAs: (**a**,**b**) control group; (**c**,**d**) mixed with PAM; (**e**,**f**) mixed with VNS-AM-AA.

SEM images of the cement paste with PAM and VNS-AM-AA are shown in Figure 12. Compared with the control group, the hydration products of the cement paste with PAM and VNS-AM-AA did not change, which were still C-S-H, Ca(OH)_2_, and AFt, but the overall morphology was changed. The control group had a flat surface and dense structure with fewer pores and cracks (Figure 12a), and doping with PAM introduced more pores (Figure 12c) and the hydration products were wrapped (Figure 12d). This is because doping with 1% PAM will react with the cations Ca^2+^, Fe^3+^, etc., to produce complexes, which wrap the surface of cement ions and hinder the migration of hydration products [42,43]. And the laminar spatial distribution network of PAM is not able to agglomerate most of the cement particles at the same time, so that the growth of the hydration products is discontinuous, and the formation of the skeleton system has more pores.

In contrast, with 0.4% VNS-AM-AA mixed with the removal of a few cracks, the pore space is relatively less, and the structure is more compact (Figure 12e). But there is also the case of wrapped hydration products (Figure 12f). It is presumed that the dosage of VNS-AM-AA is relatively small compared to PAM, so the formed complex is relatively small and the obstruction of hydration products is not as strong as PAM. On the other hand, the three-dimensional network spatial structure of VNS-AM-AA can effectively agglomerate and lock most of the cement particles, so that the generated hydration products can be grown continuously. So, the formation of the cement paste is more regular, which has a beneficial effect on the strength of the later stage and resistance to environmental erosion. Since the presence of complexes still causes a small number of cracks, VNS-AM-AA needs to be used in combination with some fillers such as fly ash or silica fume to supplement cracks or pores during actual construction.

## 4. Conclusions

In this study, a hyperbranched polymer was synthesized with NS as the inorganic component and AM and AA as organic components, which changed the linear molecular structure of conventional PAM.

(a) The synthesized VNS-AM-AA achieved the best flocculation effect when NS was selected to be 20 nm, the mass ratio of silane coupling agent VTES to NS was 0.3, the addition of NS was 0.6% of the total mass of the monomer, and the molar ratio of the organic components AM and AA was 3.5:1. The relative molecular weight of the synthesized VNS-AM-AA was about 6 million at this time. FTIR, TEM, and SEM analyses showed that the synthesized polymer was the target product, and it did not easily agglomerate in water with a three-dimensional network effect.
(b) Compatibility experiments showed that VNS-AM-AA was more compatible with the polycarboxylic acid water-reducing agent (SP), and at an SP dosing of 0.6% of cement mass, the loss fluidity in 60 min warp time was only 4 mm, with the best stability of cement paste.
(c) The anti-dispersity experiment and the flowability experiment showed that the anti-dispersity of VNS-AM-AA was better than that of PAM at a smaller dosage, but its fluidity was more sensitive to the influence of the dosage, and it could be seen that the optimal dosage of VNS-AM-AA for combined fluidity and anti-dispersity was 0.4%. Due to the deeper entanglement of cement particles, the initial setting time of cement paste was delayed by 3–6 h and the final setting time was delayed by 3–9 h after mixing in VNS-AM-AA, which were higher than those of PAM. In actual construction, VNS-AM-AA should be used with a quick-setting agent to reduce the setting time.
(d) The yield shear stress of cement mortar was tested to characterize the scouring resistance of mortar in water, and it was found that VNS-AM-AA could give cement mortar a higher yield shear stress at dosage higher than 0.4%. And the higher the yield shear stress, the less likely that the mortar would be moved by water scouring. So, VNS-AM-AA has the effect of improving the scouring resistance.
(e) Observing the microscopic morphology of the cement mortar with two AWAs after 7d, it can be found that the incorporation of PAM and VNS-AM-AA does not change the type of hydration products, but it will generate complexes to encapsulate the hydration products. Compared with more pores caused by the incorporation of PAM, the incorporation of VNS-AM-AA has relatively fewer pores and a more compact and regular structure, which is beneficial to the improvement of engineering quality.

## Figures and Tables

**Table 1:** Chemical composition of cement.

Composition	LOI	CaO	SO_2_	SO_3_	Fe_2_O_3_	MgO	Al_2_O_3_
Content/%	3.01	72.02	18.99	2.79	3.03	1.31	5.79

**Table 2:** Mix proportion of cement paste.

Types	Cement (g)	Water (g)	AWAs (wt%)	SP (wt%)
PAM	300	120	0.5~1.5	0.6
VNS-AM-AA	300	120	0.2~0.6	0.6

**Table 3:** Mix proportion of cement mortar.

Types	Cement (g)	Sand (g)	Water (g)	AWAs (wt%)	SP (wt%)
PAM	300	343	120	0.5~1.5	0.6
VNS-AM-AA	300	343	120	0.2~0.6	0.6

## Data Availability

The original contributions presented in the study are included in the article, further inquiries can be directed to the corresponding author.
